# Three years of hourly data from 3021 smart heat meters installed in Danish residential buildings

**DOI:** 10.1038/s41597-022-01502-3

**Published:** 2022-07-19

**Authors:** Markus Schaffer, Torben Tvedebrink, Anna Marszal-Pomianowska

**Affiliations:** 1 Aalborg University, Department of the Built Environment, Aalborg, 9220 Denmark; 2 Aalborg University, Department of Mathematical Sciences, Aalborg, 9220 Denmark

**Keywords:** Environmental sciences, Climate sciences

## Abstract

The now widespread use of smart heat meters for buildings connected to district heating networks generates data at an unknown extent and temporal resolution. This data encompasses information that enables new data-driven approaches in the building sector. Real-life data of sufficient size and quality are necessary to facilitate the development of such methods, as subsequent analyses typically require a complete equidistant dataset without missing or erroneous values. Thus, this work presents three years (2018-01-03 till 2020-12-31) of screened, interpolated, and imputed data from 3,021 commercial smart heat meters installed in Danish residential buildings. The screening aimed to detect data from not used meters, resolve issues caused by the data storage process and identify erroneous values. Linear interpolation was used to obtain equidistant data. After the screening, 0.3% of the data were missing, which were imputed using a weighted moving average based on a systematic comparison of nine different imputation methods. The original and processed data are published together with the code for data processing (10.5281/zenodo.6563114).

## Abstract (summary)

**Table:** 

Measurement(s)	Heat energy • Volume flow at supply • Volume flow X return temperature • Volume flow X supply temperature
Technology Type(s)	Smart heat meter
Sample Characteristic - Organism	Buildings
Sample Characteristic - Location	Aalborg

## Background & Summary

Since 25^th^ October 2020, it has been mandatory within the European Union (EU) that newly installed district heating and cooling meters are remotely readable^1^. From 2027 on, this will also apply to all meters installed before that date^1^. Such remotely readable meters, also called smart meters, have commonly an hourly or even sub-hourly temporal resolution. This high temporal resolution, combined with the fact that more than 20% of the residential sector’s heating demand in ten EU countries is covered by district heating^2^, creates a high theoretical potential for new data-driven approaches.

This high theoretical potential was confirmed by recent research, which applied clustering to smart heat meter data to identify typical consumption profiles and correlate consumption patterns and densities to occupants and building characteristics^3–9^. Furthermore, it was shown that such data could be used to derive building characteristics such as the U-value of the building envelope, the internal temperature in winter or the building’s dependency on solar gains^10,11^. Next to these application possibilities, which focus on individual buildings, the data can also be used to calibrate and validate archetype building models for urban building energy modelling^12–15^.

Besides this use for building-oriented applications, smart heat meter data also becomes relevant for district heating utility companies. Due to the increasing share of intermittently available renewable energy, the production can no longer freely follow the demand, but the demand must be adjusted according to the varying production. However, this new requirement on the operation of district heating networks necessitates in-depth knowledge of the demand side. Despite this, the smart heat meter data is currently predominantly used for billing purposes by district heating utility companies [personal communication with Aalborg Forsyning], and its inherent information remains unused.

While this literature body highlights the principle potential, the research regarding smart heat meter data is still in its infancy compared to smart electricity meter data research. To increase the awareness of smart heat meter data and to improve its availability, this work publishes the first dataset of commercial smart heat meters, including contextual information and the cleaning, interpolation, and imputation framework used for data processing. Both data and code are published at *Zenodo* (ref. ^16^) at 10.5281/zenodo.6563114. It is, however, to be mentioned that one prior work, has published aggregated information of about 43,000 smart heat meters, located in Aarhus, Denmark, to provide an overview of their district heating efficiency^17^.

The published, processed dataset contains about three years (2018-01-03 till 2020-12-31) of equidistant data without missing or erroneous values (about 0.3% of missing data were imputed) from 3,021 commercial smart heat meters installed in residential units (single-family houses, terrace houses and apartments) in Aalborg, Denmark, with a temporal resolution of one hour. The data includes cumulative values of the ‘Heat energy’, the ‘Volume flow’, the ‘Flow X temp. supply’, and the ‘Flow X temp. return’ (Table 2). Additionally, contextual data, including the type of building, the construction year and where available, the energy label is provided, which allows linking each smart heat meter to the building typologies identified by the TABULA project^18^ and statistical data about the whole Danish building stock^19,20^.

**Fig. 1:**
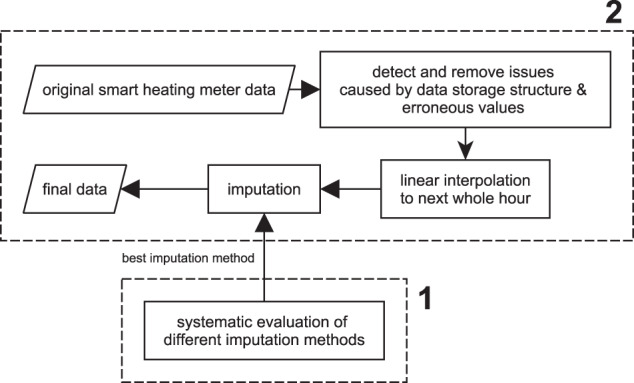
Overview of the used data processing framework. **1** indicates the part for the systematic evaluation of imputation methods, **2** is the part for data processing.

The developed data processing framework has two primary purposes (Fig. 1). First, it was used to systematically investigate single imputation methods for a representative pattern of missing data based on the normalised root mean square error (NRMSE) as an evaluation criterion. This analysis was conducted on the whole dataset for all four cumulative quantities. Thereby, the results showed that the NRMSE increases till a gap length of about 13 h, but from there on, a near-horizontal trend was seen for most investigated methods. Based on these findings, imputation by moving average was identified as the most suitable imputation method. While this evaluation currently only includes single imputation methods, it can be easily adapted to incorporate multiple imputation methods and other more advanced imputation methods. Secondly, the framework was used to detect and remove smart heat meters, which did not record any consumption during the considered period, known issues caused by the data storage process, and erroneous values. After this step, the data were linearly interpolated as the original readings had an accuracy of ±30 min around the entire hour and missing values were imputed.

## Methods

### Data acquisition, selection and anonymisation

The original dataset with data from 3,127 smart heat meters installed in Aalborg, Denmark, spanning from 2018-01-01 to 2020-12-31, was provided to the authors by the district heating utility company (Aalborg Forsyning) in Aalborg. Fellow researchers wishing to gain access to this should contact the corresponding author, who can establish the contact with the district utility company. Due to the below-described anonymisation, the customers from which this data originated were not notified. The district heating utility company collected the data for billing purposes and has selected the data for this work at random under the condition that not more than 10% of the data are missing for the selected three-year period and under the precondition that the data should be from single-family houses. The data were supplied in column organised comma-separated files split by the postal codes of the building and further split so that each file has a maximum of five million rows. The district heating utility company did not process the data.

Next to these data, the authors could obtain contextual data based on publicly available information^20,21^ based on the smart meter addresses that the district heating utility company supplied. This contextual data includes the year of construction, the type of building and where available the energy label. This information is published along with the smart heat meters’ data and enhances the usability of the presented dataset, as described in ‘Usage Notes’. Moreover, based on the information about the type of the building type, the authors could identify that despite prior belief, not all smart heat meters are installed in single family-houses but also in other building types, as shown in Table 1. The building could not be unambiguously identified for about 3.1% of the smart heat meters. Reasons for this can be that there are, e.g., multiple buildings within one address. The overall deviation from the expected use, solely single-family houses, can not be explained with certainty. However, it is hypothesised that this is caused by outdated information at the district utility company, i.e., if a building usage changes or human error when the data were selected. Consequently, it was decided to consider from here on only the 3,022 smart heat meters that could be clearly linked to residential units. The published raw data includes however the data of all 3,127 smart heat meters.

**Table 1:** Distribution of building types in which the smart heat meters of the raw data are installed. Identification is based on data of the danish ‘Building and Housing Register (BBR)’^21^. Unclear refers to units where the usage type could not be clearly identified. n = 3,127 smart heat meters.

Type	Number	Percent
Single-family house	2,460	78.7
Terraced house	474	15.2
Apartment	88	2.8
Non-residential	8	0.3
Unclear	97	3.1
	**3,127**	**100**

The data of the remaining 3,022 smart meters were collected by commercial smart heat meters from the manufacturer ‘Kamstrup’ (‘MULTICAL 402’ and ‘MULTICAL 403’^22,23^), which is, to the best of the authors’ knowledge, the most common manufacturer of smart heat meters used in Denmark. This is also confirmed by one prior study, which published aggregated data of about 43,000 smart heat meters in Aarhus, Denmark, from the same manufacturer and type^17^. All in the raw data included variables and, where applicable, their reporting resolution are shown in Table 2. Further, it is to be noted that the in Table 2 shown reporting resolution corresponds to the resolution when the reading is transmitted to the district heating utility company, whereby the values are rounded to reduce bandwidth [personal communication with Aalborg Forsyning]. The actual measurement accuracy of the smart heat meters used is higher than the reporting resolution^22,23^. The smart meters transmit their data every full hour with an accuracy of ±30 min. The data transmitted reflects the measurement at this time, i.e. a reading transmitted at 13:15 includes for cumulative values, all consummation up until 13:15 [personal communication with Aalborg Forsyning]. The unique meter and customer identifier were anonymised by replacing them with random integers to prevent particular customers from being identified. Furthermore, the files were renamed with random numbers to remove the postal code information. This anonymisation does not influence the usability of the data significantly.

**Table 2:** Columns of the raw and processed data, including their reporting resolution. *The processed data’s reading time refers to the new equidistant time resulting from the described processing. The columns ‘raw data’ and ‘processed data’ indicated which column is included in the provided raw data and the final processed data. cum. = cumulative, inst. = instantaneous, max. = maximum.

Original column name	English name	Raw data	Processed data	Description	Type	Resolution
CustomerID	Customer id	✓	✓	Identification of the customer	—	—
MeterID	Meter id	✓	✓	Identifier for the meter	—	—
Aflæsningstidspunkt	Reading time	✓	✓*	Reading timestamp as date and time	—	1 min
Energi 1 Varmeenergi	Heat energy	✓	✓	Heat energy deposited	cum.	1 kWh
Enhed	Unit	✓	✓	Unit for ‘Heat energy’ - kWH	—	—
Volumen 1	Volume flow	✓		District heating water volume passed through the meter - measured at the supply	cum.	0.01 m^3^
Enhed2	Unit2	✓		Unit for ‘Volume flow’	—	—
Timetæller	time counter	✓	✓	Number of hours the meter has been in operation	cum.	1 h
Enhed3	Unit3	✓		Unit for time counter - hours	—	—
Energi 8	Flow X temp. supply	✓	✓	Volume flow (supply) × supply temperature	cum.	1 m^3^ °C
Enhed4	Unit4	✓		Unit for ‘Flow X temp. supply’ – m^3^ °C	—	—
Energi 9	Flow X temp. return	✓		Volume flow (supply) × return temperature	cum.	1 °C
Enhed5	Unit5	✓		Unit for ‘Flow X temp. return’ –m^3^ °C	—	—
Temperatur 1	temperature 1	✓		Supply flow temperature	inst.	1 °C
Enhed6	Unit6	✓		Unit for ‘temperature 1’ - °C	—	—
Temperatur 2	temperature 2	✓		Return flow temperature	inst.	1 °C
Enhed7	Unit7	✓		Unit for ‘temperature 2’ - °C	—	—
Flow 1	flow 1	✓		Current supply flow through the meter	inst.	0.01 m^3^
Enhed8	Unit8	✓		Unit for ‘flow 1’ – m^3^	—	—
InfoKoder	info codes	✓		Meter status - is not in use and thus it is not complete nor correct	—	—
Effekt 1	effect 1	✓		Current deposited heating effect - only recorded for meter type MULTICAL 403	inst.	0.1 kW
Enhed9	Unit9	✓		Unit for ‘effect 1’ - kW	—	—
Maks.-effekt 1	max. effect 1	✓		Maximum deposited heating effect during it lifetime - only recorded for meter type ‘MULTICAL 403’	max.	0.1 kW
Enhed10	Unit10	✓		Unit for ‘max. effect 1’ - kW	—	—
Målertype	Meter type	✓		Type of smart meter	—	—
RoundedReadTime	—	✓		‘Reading time’ rounded to the next whole hour	—	—

Given the temporal resolution of approximately one hour, the instantaneous values, which are spot measurements at the time when the meter transmits the hourly reading, are less informative and are thus not considered further. However, they are included in the provided unprocessed raw dataset. Consequently, only the four cumulative quantities ‘Heating energy, Volume flow’, ‘Flow X temp. supply’ and ‘Flow X temp. return’ (Table 2) were considered from here on.

### Data cleaning

For the following described data cleaning process, the following three aspects were considered:

- Smart meters not in use during the three years
- Issues caused by the data structure respectively the data storing process at the district heating utility company
- Values not following the cumulative trend of the data

Outliers were deliberately not considered as the commercial smart heat meters used to collect the data have a built-in quality control^22,23^. Consequently, outliers are more likely caused by faulty operation of the heating installation caused by technical or human error than by measurement error. Thus removing/replacing such values would eliminate potentially useful information.

#### Detection and removal of unused smart meters

As the first step of the cleaning process, it was validated that the smart heat meters were used during the three years of the measurement period. To be considered in use, each cumulative value has to change at least once during the three years. This analysis identified no smart heat meter which was not in use, and consequently, no data was removed.

#### Issues caused by the data storage detection & removal

In the second step of the screening process, known issues arising from the data storing process at the district heating utility company were resolved. After initial analyses and personal communication with the district heating utility company [personal communication with Aalborg Forsyning], the first identified issue was that the smart heat meters could be temporarily incorrectly assigned to two customers. This incorrect allocation is caused by human error, as the customer must be manually registered when the meter is installed. This issue was resolved by removing smart meters with more than one customer assigned - this removed data of one smart meter so that 3,021 (79,584,072 rows) remained.

The second identified problem concerns Denmark’s daylight-saving time (DST). The readings of the smart heat meters were stored in local time without time zone information. Consequently, a value measured between 2:00 - 2:59 CET or 2:00 - 2:59 CEST on the day when the DST ends is stored identically. Thus, a reading at 2:10 CEST and 2:10 CET on that day would appear as two simultaneous readings even though they are one hour apart. Moreover, the following situation can arise when the DST ends: One smart meter reading is received at 2:20 CEST, and the subsequent measurement is obtained at 2:10 CET. Accordingly, the values are stored as 2:20 and 2:10, and the second later reading now wrongfully appears to be measured earlier. Assuming there is no error, one could order the readings according to the size of the cumulative quantities to resolve these issues if both readings exist. However, this becomes impossible if one reading is missing. Thus, it was decided to remove all measurements registered between 2:00 and 2:59 on the day when the DST ends. This data cleaning removed 17,294 (0.02%) rows (79,566,778 rows remained).

#### Erroneous value detection & removal

The third step of the screening process was to detect erroneous values by utilising the cumulatively of the quantities. Therefore, it was evaluated if each value is equal to or larger than its predecessor. If that was not true, the whole data record at the time of the value and its predecessor was removed. The entire record was removed as all four cumulative quantities are results of each other, and thus it is likely that one erroneous quantity influences the reported values of the other three. The value and its predecessor were removed, as it cannot be directly determined if one is too small or the other is too large. This screening method could be applied as potential erroneous values result from transmission errors rather than measurement errors, as such would be detected by the quality control of the smart heat meter. This screening identified 6 values in 4 different data rows, and thus 8 data rows were removed (79,566,770 rows remained).

### Interpolation

As the commercial smart heat meters have a temporal accuracy of ±30 min around the full hour of the data were not equidistant. Consequently, the existing measurements were interpolated to the next full hour using linear interpolation. For each smart heat meter’s first and last observation, linear extrapolation was used if necessary. After the interpolation, it was found that while the data of each smart meter should span from 2018-01-01 to 2020-12-31, some data starts as late as 2018-01-02 at 13:00. Consequently, it was decided to remove data before 2018-01-03 so that all data begin simultaneously without extensive extrapolation. For the same reason, all data after the 2020-12-31 23:00 were removed (79,082,699 rows remained).

### Imputation

After applying the above-described Data cleaning and interpolation, about 0.3% of the remaining 3,021 smart heat meter data were missing. For all missing values, it is known that the following data points also include the consumption over the missing period. This is known as missing data results from the transmitting process from the smart heat meter to the utility company, i.e. at the smart meter itself, no data is missing. Based on the findings of the systematic evaluation of different imputation methods (‘Systematic imputation comparison’), it was decided to impute the missing data using imputation by weighted moving average with a symmetric window size of 48 (48 previous and following values) and a linear weighting. Each of the four cumulative quantities was imputed separately for each smart meter. However, as this imputation method does not lead to results that inevitably follow the cumulative trend of the data, as not the cumulative values were used but the non-cumulative demand calculated according to Eq. 1, where *d*_*t*_ is the demand value at time instance *t*, and *c*_*t*_ respectively *c*_*t-*1_ are the cumulative values at time instance *t* and *t*-1.

1 $${d}_{t}={c}_{t}-{c}_{t-1}$$

To utilise the from the cumulative values derived knowledge of “how much” of each quantity is missing for each gap, the imputed values were scaled. This concept was used before for the imputation of smart electricity meter data^24^ and ensures that the imputed data follows the cumulative trend. For this scaling first the real amount of missing quantity was calculated based on Eq. 2, where *C*_*i*_ is the amount of quantity missing for gap *i* which spans from *t*_*i*_ to *t*_*i*_ + *k*_*i*_, ${o}_{{t}_{i}-1}$ is the last observed cumulative value before and ${o}_{{t}_{i}+{k}_{i}+1}$ is the first observed cumulative value after the gap *i*.

2 $${C}_{i}={o}_{{t}_{i}+{k}_{i}+1}-{o}_{{t}_{i}-1}$$

After this, the imputed demand values were scaled for each gap using Eq. 3 where ${p}_{{t}_{i}}$ is the scaled imputed demand value at the time *t*, ${p}_{{t}_{i}}^{{\prime} }$ is the imputed demand value at the time *t*, *C*_*i*_ is actual amount of quantity at the time *t*, all of the gap *i*. Hereby it is to be noted, that a gap *i* spanning from *t*_*i*_ to *t*_*i*_ + *k*_*i*_ in the cumulative values introduces a gap from *t*_*i*_ to *t*_*i*_ + *k*_*i*_ + 1 in the demand values. Before Eq. 3 was applied, any imputed negative value (${p}_{{t}_{i}}^{{\prime} } < 0$), thus physical impossible value, was set to zero.

3 $${p}_{{t}_{i}}={p}_{{t}_{i}}^{{\prime} }\cdot \frac{{C}_{i}}{{\sum }_{{s}_{i}={t}_{i}}^{{t}_{i}+{k}_{i}+1}\left({p}_{{s}_{i}}^{{\prime} }\right)}$$

If ${\sum }_{{s}_{i}={t}_{i}}^{{t}_{i}+{k}_{i}+1}\left({p}_{{s}_{i}}^{{\prime} }\right)=0$ and thus Eq. 3 is not defined, Eq. 4 was used instead for scaling the imputed values.

4 $${p}_{{t}_{i}}={p}_{{t}_{i}}^{{\prime} }+\frac{{C}_{i}}{{k}_{i}+1}$$

After the scaling, the scaled imputed demand values were used to replace all missing values and afterwards, the complete demand values were used to calculate the cumulative values.

## Data Records

The data accompanying this work is available on the repository *Zenodo* (ref. ^16^) at 10.5281/zenodo.6563114 under Commons Attribution 4.0 International (CC-BY 4.0) license (https://creativecommons.org/licenses/by/4.0). There two folders named ‘01_Data’ and ‘02_R_code’ can be found. Within the folder ‘01_Data’ the data is organised in the folder ‘01_anonymised_raw_data’, ‘02_processed_final_data’, ‘03_context_data’, and ‘04_data_visualisation’. In the first folder, the anonymised original unprocessed data (of all 3,127 smart heat meters) are available as 25 .csv files split by the anonymised postal codes of the buildings and a maximum length of 5 million rows, following the structure in which the authors obtained the data. The complete equidistant data from the described processing steps are available in the second folder as one .csv file per smart heat meter. The third folder includes one .csv file that specifies for each smart meter building the type of building (according to Table 1), the construction year, and the energy label, if available. The unique meter identifier can link this data to the consummation data. All .csv files use a comma as a separator and a dot as a decimal separator. The last folder (‘04_data_visualisation’) includes additional figures as .png files visualising the demand pattern of the presented data s described in ‘Usage Notes’. In addition, a.pdf file named ‘05_data_description’ is included in the folder ‘01_Data’, which describes both the original and processed data, similar to Table 2.

## Technical Validation

Various tests and analyses were performed to ensure the presented dataset’s reliability and quality.

### Rerun erroneous value detection

The above in ‘Erroneous value detection & removal’ described detection and removal of erroneous values was re-run on the final cleaned, interpolated, and imputed dataset. Thereby no values were identified, and the dataset remained unchanged, demonstrating that the imputed values follow the data’s cumulative trend.

### Interpolation

**Fig. 2:**
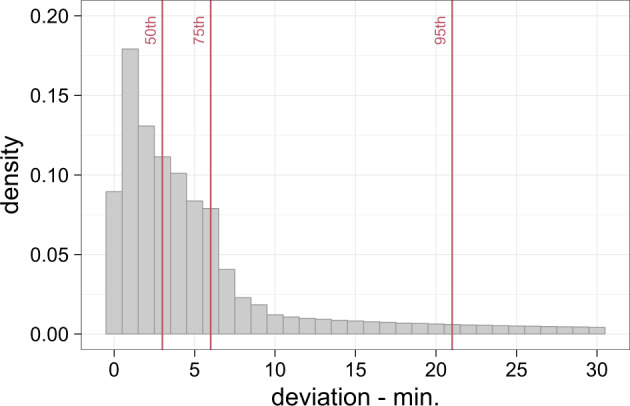
Absolute temporal deviations of meter readings to the next full hour, including the 50^th^, 75^th^, and 90^th^ percentile. Based on the data after the ‘Data cleaning’ process. n = 3,021 smart heat meters.

The accuracy of the linear interpolation used to interpolate existing smart heat meter reading to the next full hour cannot be straightforwardly shown as the real values are unknown. However, an analysis of the temporal deviation from the original values to the next full hour (Fig. 2) showed that 50% of the data points deviate ≤3 min and 75% of the data points deviate ≤6 min. While this gives no direct indication of the accuracy, the relatively small temporal deviation indicates that the error caused by the linear interpolation should be minor. However, further investigations with, e.g., higher temporal resolution data, are necessary to estimate the accuracy of this interpolation step more precisely.

### Systematic imputation comparison

Currently, the literature regarding the imputation of smart heat meter data is sparse. Only one work^9^ investigated various simple single univariate imputation methods on the data of 10 smart heat meters and concluded conclude that linear interpolation leads to the best results for cumulative quantities. Because of the small sample size, it was decided to systematically evaluate different imputation methods on the whole dataset to identify the most suitable one.

The main idea of the systematic evaluation conducted in this work is to remove known data from the dataset and impute these artificial gaps with various methods. Therefore, the artificially created gaps represent the average gaps per meter in length and amount. The imputed values are then compared against the known real values to assess the accuracy of each method. In following these steps are described in detail.

#### Analysis of missing data & artificial gap generation

**Fig. 3:**
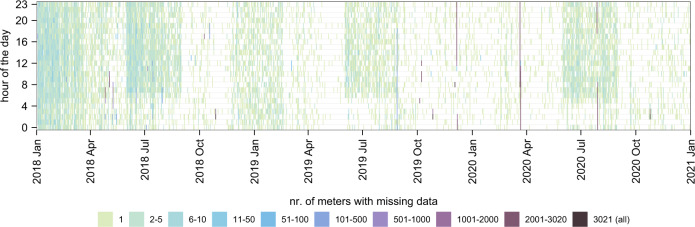
Temporal distribution of smart meters with simultaneous missing data. Based on the data after the ‘Data cleaning’ and ‘Interpolation’ process. n = 3,021 smart heat meters.

After the ‘Data cleaning’ and ‘Interpolation’ process, the distribution of missing values in the dataset was investigated to determine if the data were missing at random. As shown in Fig. 3, there seem to be periods such as December 2018 to February 2019 where data are more likely to be missing for multiple meters simultaneously. The reason for this pattern could not be determined. It was hypothesised that the pattern is correlated with specific weather conditions, but this could not be confirmed. However, given the low number of smart meters which have missing data in these periods, it is possible that random effects can cause these, and thus these patterns are considered insignificant. Next to this, there are a few points where most of the meters have missing data. An error in the transmitting network used to receive the data most likely causes these periods. Consequently, it was concluded that missing data occurred at random throughout the whole dataset.

The gap lengths and their average frequency per smart meter were next calculated next. The results are shown in Table 4, whereby the frequency averaged per meter is rounded up to the next integer. Thus, this leads to an overrepresentation of particularly the longer gaps (<3) but simplifies the subsequent step to create artificial gaps, and thus, this was seen as reasonable.

This gap length-frequency distribution was subsequently used to create artificial gaps in the data of each smart meter after the ‘Data cleaning’ and ‘Interpolation’ procedures. Thereby the gaps were randomly distributed within the data of each smart heat meter, and all four cumulative quantities were removed simultaneously to mimic the structure of the actual gaps. Existing missing data were ignored (i.e., artificial gaps can be introduced at a time instanced where data are already missing). The artificial gaps were introduced in the cumulative values, and if needed, demand values were calculated after that.

#### Imputation methods

As this is the first time that different imputation methods for smart heat meter data have been investigated to such an extent, it was decided to use an application-oriented approach and to focus on standard “out of the box” imputation methods for the systematic evaluation. Consequently, the focus fell on commonly used and (in R^25^) readily available single imputation methods. Additionally, it was decided to focus on computationally inexpensive methods due to the low percentage of missing data (0.3%) and the relatively large amount of data. The ‘CRAN Task View’ for missing data^26^, which gives an overview of relevant R packages for handling missing data, was used to identify appropriate imputation methods, respectively R packages. Based on this overview, two highly used and cited R packages, including various imputation methods, were identified and used for inspiration. VIM^27^, as a general-purpose package for imputation, and imputeTS^28^, as a dedicated package for time series data. Because of the focus on computationally inexpensive methods, expensive methods such as the ‘iterative robust model-based imputation (IRMI)’ were not considered. Besides the methods from these two R packages, the increasingly popular method of imputation using random forest as implemented in the R package missRanger^29^ was selected. (This is a faster^26^ implementation based on the missForest^30^ package.) Moreover, given the nature of the data, imputation using a spline interpolation was seen as a feasible and applicable method. Thus, both a monotone Hermite spline and a spline were investigated. An overview of all investigated methods, an abbreviated name and a short description of each method is given in Table 3. For a detailed description, the interested reader is referred to the previously cited literature. The following outlines how the selected imputation methods were applied to the smart heat meter data and where necessary methods are further specified.

**Table 3:** Imputation methods used for the systematic comparison.

Abbreviation	Description	Value used for imputation	R-command
Linear	Linear interpolation	cumulative	imputeTS::na_interpolation^28^
MonoH.FC	Monotone Hermite spline according to the method by Fritsch and Carlson^31^	cumulative	stats::splinefun^25^
Moving avg.	Moving average imputation with a symmetric window size of 48 (48 previous and 48 following observations) with a linear weighting	demand	imputeTS::na_ma^28^
Constant	Imputation by a constant value leads, due to the scaling, to a constant demand which fits the amount of missing data per gap	demand	imputeTS::na_replace^28^
Random	Interpolation with an random value with the lower and upper bound determined by the minimum and maximum of the existing data	demand	imputeTS::na_random^28^
Akima	Akima spline^32^	demand	akima::aspline^35^
Kalman	Kalman Smoothing on a structural model fitted by maximum likelihood	demand	imputeTS::na_kalman^28^
Hotdeck	Sequential hotdeck algorithm - if no suitable donor is found a random donor is used	demand	VIM::hotdeck^27^
RF	Chained random forest imputation based on the idea of missForest^30^	demand	missRanger::missRanger^29^

**Table 4:** Total count and over all smart meter averaged frequency, with the frequency rounded up to the next integer. Based on the data after the ‘Data cleaning’ and ‘Interpolation’ process. n = 3,021.

gap length	count	avg. frequency per meter
1	34,943	12
2	14,027	5
3	6,720	3
4 to 29	1,343; 1,308; 894; 867; 854;	1
	790; 704; 600; 611; 773;	
	1,098; 779; 739; 138; 217;	
	229; 129; 137; 153; 158;	
	165; 167; 175; 265; 454; 11	

##### Linear and MonoH.FC

These imputation methods obey the data’s cumulative trend, and thus the cumulative values were used for each quantity. Each quantity was furthermore imputed separately for every smart meter. For the monotone Hermite spline, the Fritsch-Carlson method^31^ was used.

##### Constant, Random, Moving avg., Akima, Kalman

Again, each quantity was imputed separately for every smart meter for these five imputation methods. However, as these imputation procedures do not necessarily lead to results that comply with the cumulative trend of the data, the non-cumulative demand values, including the proposed scaling procedure as outlined in ‘Imputation’ were used. The weighted moving average imputation used a window width of 48 and a linear weighting. The width of the moving average window and the weighting were based on an initial investigation where windows sizes of 4, 8, 12, 24 and 48 and linear, exponential, and no weighting were tested. An Akima spline^32^ was used for the spline, based on an initial investigation where an Akima spline^32^ was compared against a natural spline. (The codes to perform these investigations are published as part of the accompanying code.) The imputation by a constant value leads due to the applied scaling (‘Imputation’) to the same results as, for example, imputation by last observation carried forward or imputation by mean, which is a constant demand “fitting” the amount of missing quantity per gap.

##### Hotdeck and RF

For these two approaches for each quantity and smart heat meter, the following variables were supplied to the imputation algorithm:

1. demand value of the to be imputed quantity (calculated according to Eq. 1)
2. 24 h leading and lagged demand value of the to be imputed quantity
3. temperature and global solar irradiation of a meteorological station about 20 km north of Aalborg (closest public weather station)^33^.

After the imputation, the demand value of the imputed quantity was scaled using the scaling procedure outlined in ‘Imputation’. It is to be noted that during an initial investigation also, a hierarchic approach was tested where before imputed quantities were additionally supplied to the imputation algorithm for subsequent quantities. This investigation was conducted for two orders, ‘heating Energy’, ‘Flow X temp. return’, ‘Flow X temp. supply’, and ‘Volume flow’ and its reverse order. However, this did not significantly increase the accuracy while the computational time increased. (The codes to perform these investigations are published as part of the accompanying code).

#### Evaluation criteria

The Normalized Root Mean Square Error (NRMSE) was used to evaluate the various imputation methods calculated based on Eq. 5 where *p*_*t*_ is the imputed value at time instance *t*, *o*_*t*_ the real observed value at time instance *t*, and $\mathop{o}\limits^{\_}$ is the mean of the real observed values. It is to be noted that the NRMSE is not defined when $\mathop{o}\limits^{\_}=0$. In this case, the NRSME is set to 0 if the result beneath the square root is also 0, i.e., all real and imputed values are 0.

5 $$NRMSE=\frac{\sqrt{\frac{{\sum }_{t=1}^{n}{({p}_{t}-{o}_{t})}^{2}}{n}}}{\mathop{o}\limits^{\_}}$$

Furthermore, the NRMSE was not only calculated based on all gaps but also separately for all different individual gap lengths. This detailed breakdown of how the uncertainty caused by the imputation changes with the gap length was inspired by the fact that current literature regarding smart heat meter data shows varying exclusion criteria regarding the longest allowed gap length^7,9,11,13^. Thus, this detailed evaluation should help fellow researchers to decide if data of meters that include gaps longer than a certain threshold should be excluded due to the uncertainty caused by imputation.

**Fig. 4:**
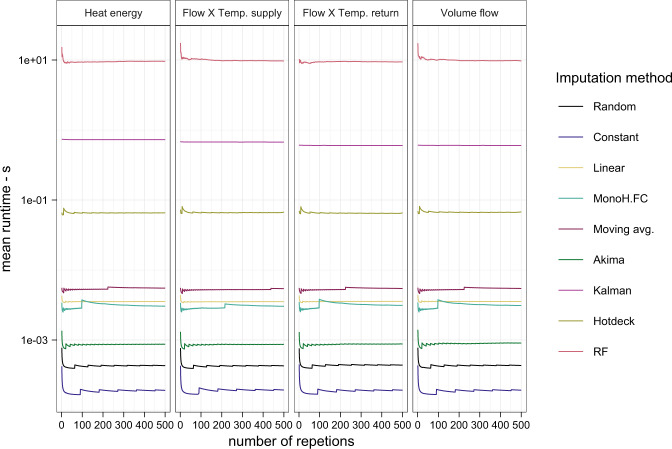
Mean runtime in seconds for each imputation method, successively including more repetitions to validate that the mean is close to the true mean. Abbreviations according to Table 3. Based on the data after the ‘Data cleaning’ and ‘Interpolation’ process. n = 3,021 smart heat meters.

In addition to this qualitative assessment of the imputation results, the average runtime for each imputation method was recorded to measure its computational efficiency. The runtime was measured in a separate process by imputing the data of one random meter sufficiently often to obtain a representative mean runtime. To ensure that the obtained mean is close to the true mean, the convergence of the result was visually inspected by consecutively including more repetitions (Fig. 4). The runtime was measured on a laptop with an Intel Core i7-10850H processor and 32GB memory.

#### Results

**Fig. 5:**
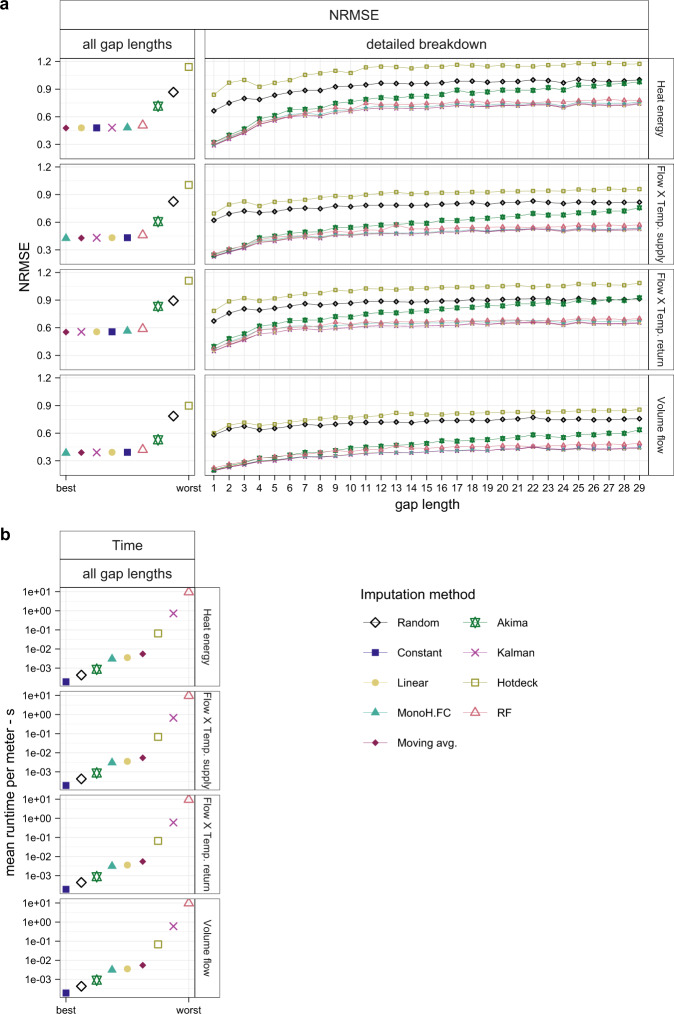
Results of the systematic evaluation of different imputation methods. Abbreviations according to Table 3. (**a**) shows the results for the NRMSE, (**b**) for the mean runtime per meter. ‘all gap lengths’ indicates that all artificial gaps were used for the imputation and the subsequent evaluation, while for the detailed breakdown, the NRMSE was calculated only for gaps with the given length. Based on the data after the ‘Data cleaning’ and ‘Interpolation’ process. n = 3,021 smart heat meters for the NRMSE and n = 500 repetitions for the mean runtime.

Analysing first the results of the NRMSE (Fig. 5a), the results indicate three different groups of imputation methods overall. First, six methods (‘Constant’, ‘Linear’, ‘MonoH.FC’, ‘Moving avg.’, ‘Kalman’, and ‘RF’) lead to nearly identical results and trends for all quantities, whereby ‘RF’ shows slightly worse results than the other methods. These six methods also show the same trend for the detailed breakdown by the gap length. Their NRMSE increases sharply till a gap length of four, from where the increase flattens before it is nearly horizontal from a gap length of 13 on. The second group, the method ‘Akima’, shows the same trend as the previously mentioned methods for short gap lengths, but the increase in NRMSE does not decrease as strongly, and the NRMSE continues to rise till the most extended gap length. Thus ‘Akima’ leads to higher NRMSEs for long gaps. The third group, ‘Random’ and ‘Hotdeck’, show minor variation between the different gap lengths and significantly higher NRMSE than the other methods. Thereby ‘Hotdeck’ leads to even worse results than ‘Random’.

The trends are similar between the four quantities, but the ‘Volume flow’ leads to the slightly lower NRMSE and the ‘Flow X Temp. return’ to slightly higher NRMSE than the other two quantities. The best method considering all gap lengths is either ‘MonoH.FC’ for the ‘Volume flow’ and the ‘Flow X Temp. supply’, or ‘Moving avg.’ for the other two quantities. However, the difference between the best five methods (’Constant’, ‘Linear’, ‘MonoH.FC’, ‘Moving avg.’, ‘Kalman’) is only marginal. It is further to be noted that the NRMSE considering all gap lengths, must not lie within the bounds of the detailed breakdown by gap length, as it is not a weighted average but an NRMSE calculated for all imputed values independent of their gap length. An example of this is the ‘Hotdeck’ imputation for the ‘Volume flow’.

For the mean runtime per smart heat meter (Fig. 5b), the results show, as expected, no difference between the four investigated quantities. Nevertheless, the methods differ significantly from each other, with the slowest method, ‘RF’, taking more than 93,000 times longer than the fastest method, ‘Constant’. Overall six methods, (’Constant’, ‘Random, ‘Akima’, ‘MonoH.FC’, ‘Linear’, ‘Moving avg.’) take around or less than 1 × 10^−3^ s. ‘Hotdeck’, the next slowest method, takes with ~0.05 s, already considerably longer, but is still about six times faster than ‘Kalman’ with ~0.3 s. ‘RF’ is, as mentioned, by far the slowest method with ~10 s per meter and quantity.

From these results it can overall be concluded that not one imputation method shows clearly superior results. Five methods (’Constant’, ‘Linear’, ‘MonoH.FC’, ‘Moving avg.’, ‘Kalman’) show overall comparable results. It is to be highlighted that even the elementary method ‘Constant’ leads in combination with the proposed scaling to competitive results. In contrast, ‘Hotdeck’ leads to even worse results than imputation by a random value (’Random’). Given the detailed breakdown by gap length, it further seems that the gap length for gaps longer than 13 does not significantly influence the uncertainty, while the difference between a gap length of one and four is significant. Thus, excluding data with a gap length longer than a certain threshold only increases accuracy by decreasing the share of long gaps. When these results are used to evaluate the uncertainty caused by the imputation of the ‘real’ missing data, it must be kept in mind that the used gap-frequency pattern overestimated the occurrence of gaps longer than three; thus, the real overall uncertainty will be likely lower than the results indicate. In terms of runtime, the results indicate a significant relative difference between the methods, whereby the absolute difference is only substantial for ‘RF’. Further, for the runtime, it must be considered that this depends not only on the method but also on its implementation in R and could thus deviate for other software or if other R packages are used.

Overall, ‘Moving avg.’ seems to lead to the best results as it is the best method for two quantities and the second-best method for the other two quantities while being computationally efficient.

Finally, it must be noted that ‘RF’ was used with settings focused on computational efficiency. Thus, it is possible that better results could have been achieved with ‘RF’ at the cost of increasing the computational cost. However, given that ‘RF’ was already the computational most costly method and that only ∼0.3% of the data were missing, this was deemed unreasonable.

## Usage Notes

The presented complete, cleaned, and equidistant dataset can be used to advance the research regarding smart heat meter data. Inspiration for further research can be drawn from the existing literature briefly outlined in the ‘Background & Summary’ section.

**Fig. 6:**
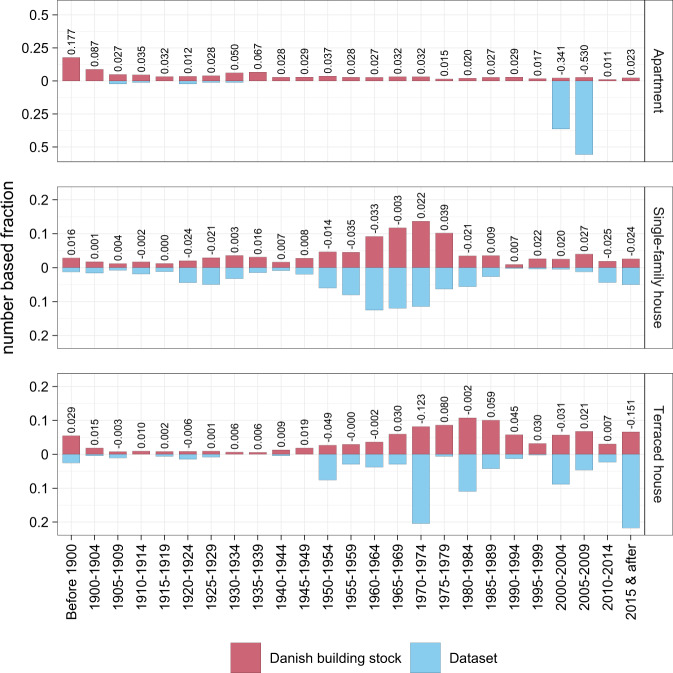
Comparison of the distribution of construction years of the smart heat meter’s buildings (n = 3,021) against the whole Danish building stock^19^. Construction year periods are taken over from Statistics Denmark. The number indicates the deviation between the data of Statistic Denmark to the presented data rounded to three digits.

As mentioned in ‘Data acquisition, selection and anonymisation’ and ‘Data Records’, next to the presented data, also contextual data are provided to enhance the usability. This data includes the year of construction, the building type and, where available, the energy label. The provided year of construction allows to compare the presented data against the whole Danish building stock, based on the construction periods defined by Denmark Statistic^19^ (Fig. 6). Based on this comparison, it can be said that the data is highly representative of the whole danish building stock for single-family houses. However, for the terraced houses, the representatives decrease, and for the apartments, it can be seen that the data is not representative as most of the buildings are from two construction year periods only.

**Fig. 7:**
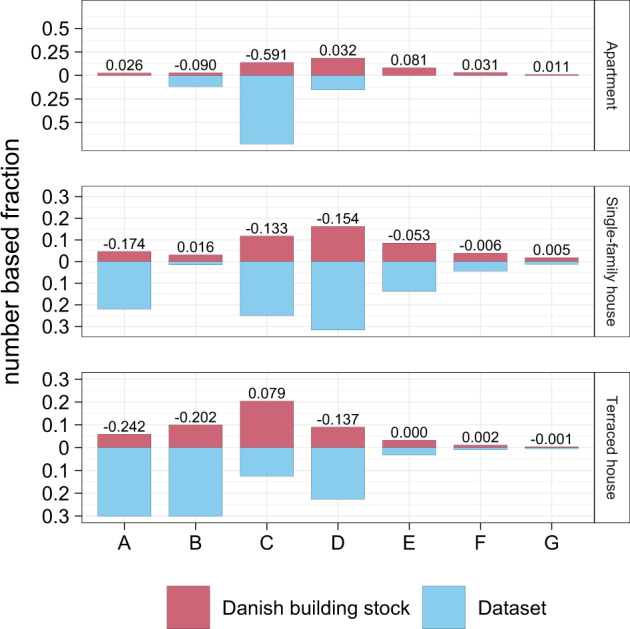
Comparison of the energy label of the smart heat meter’s buildings (where available - n = 1,283) against the whole Danish building stock for all construction years^20^. The number indicates the deviation between the data of Statistic Denmark to the presented data rounded to three digits.

In addition to the year of construction, the contextual data provides, where available, the energy label of the respective building. The energy labels allow for classifying the expected energy consumption of the buildings. Further, the data can be again compared against statistical information to assess the representatives of the buildings from which the smart heat meter data origins. Figure 7 shows such a comparison against the entire building stock for all construction years^20^. Thereby, as expected, the same overall trend as for the construction periods can be seen. The energy labels of the single-family houses have a distribution close to the whole building stock. It is, however, also visible that the label ‘A’ is over-represented. This over-representation is caused by the fact that new and newly renovated buildings are more likely to be equipped with a smart heat meter than older or not renovated buildings.

Lastly, the provided year of construction allows, in combination with the type information, to link each smart heat meter to representative exemplary Danish buildings as defined by the TABULA project^18^. From the TABULA project detailed information about building constructions, HVAC systems, and energy consummation can be obtained.

Next to the presented data, the used multistep cleaning and imputation framework and, in particular, the systematic imputation framework presented in ‘Systematic imputation comparison’ can also be applied to other smart heat meter data or data of similar nature. Consequently, this can facilitate an easy screening and imputation process for subsequent research which uses other than the presented data. Additionally, the used imputation framework can be easily adapted to incorporate multiple imputation methods and other more advanced imputation methods.

To ease the understanding of the data, Figures visualising the *z-normalised* demand of the four cumulative quantities in different temporal resolutions are provided in the used repository in the folder ‘01_Data/04_data_visualisation’. The code to create these figures is also provided.

## Data Availability

The code used for the data processing, the technical validation and the calculation and visualisation of the *z-normalised* demand is available on the repository *Zenodo* (ref. ^16^) at 10.5281/zenodo.6563114 under Commons Attribution 4.0 International (CC-BY 4.0) license (https://creativecommons.org/licenses/by/4.0). All code is based upon the R programming language version 4.05^25^. The upload is structured in two folders named ‘01_Data’ and ‘02_R_code’. Within the folder ‘02_R_code’, the code is structured into three folders: ‘01_main’, ‘02_technical_validation’, and ‘03_additional’. The first folder contains the code necessary to process the original data as described in ‘Methods’ to obtain the complete equidistant dataset. The second folder contains the code used for the analyses described in ‘Technical Validation’ including their initial investigations. The third folder contains the code necessary for the in ‘Usage Notes’ mentioned calculation and visualisation of the *z-normalised* demand and the mentioned initial investigations of imputation methods. Each folder contains its own R project, the related code files, and a “readme.pdf” file describing the purpose of each R code file. The user must create a free API key to the weather data used for‘ Hotdeck’ and ‘RF’ imputation methods. The steps, therefore, are described here: https://confluence.govcloud.dk/display/FDAPI/User+Creation, the R code to obtain the weather data is provided. The R package renv^34^ was used for package management for each R project. If the user has R and the package renv installed, the command renv::restore() installs all necessary R packages in the applied version. If the user wishes not to use the package renv, the file “renv.lock”, which can be opened with a common text editor, includes a list of all used packages, their version, and their dependencies.
